# Biomechanical Comparison of Three Different Intramedullary Nails for Fixation of Unstable Basicervical Intertrochanteric Fractures of the Proximal Femur: Experimental Studies

**DOI:** 10.1155/2018/7618079

**Published:** 2018-12-11

**Authors:** Dae-Kyung Kwak, Won-Hyeon Kim, Sung-Jae Lee, Sang-Hyun Rhyu, Chul-Young Jang, Je-Hyun Yoo

**Affiliations:** ^1^Department of Orthopaedic Surgery, Hallym University Sacred Heart Hospital, Hallym University School of Medicine, Anyang, Republic of Korea; ^2^Department of Biomedical Engineering, Inje University, Kimhae, Republic of Korea

## Abstract

**Objectives:**

This biomechanical study was conducted to compare fixation stability of the proximal fragments and their mechanical characteristics in proximal femur models of unstable basicervical IT fractures fixed by cephalomedullary nailing using 3 different types of the femoral head fixation.

**Methods:**

A total of 36 composite femurs corresponding to osteoporotic human bone were used. These specimens were fixed with Gamma 3 (hip screw type; group 1) in 12, Gamma 3 U-blade (screw-blade hybrid type; group 2) in 12, and proximal femoral nail antirotation-II (helical blade type; group 3) in 12, respectively, and an unstable basicervical IT fracture was created by an engraving machine. After preloading and cyclic loading, the migration of the proximal fragment according to 3 axes was assessed by the stereophotogrammetric method and the migration of screw or blade tip within the femoral head was measured radiographically. Next, the vertical load was continued at a speed of 10 mm/min until the construct failure occurred. Finite element analysis was additionally performed to measure the stress and compressive strain just above the tip of screw or blade within the femoral head.

**Results:**

The rotational change of the proximal fragment according to the axis of screw or blade was much greater in group 1 than in groups 2 and 3 (p=0.016 and p=0.007, respectively). Varus collapse was greater in group 3 than in group 2 (p=0.045). Cranial and axial migration of screw or blade within the femoral head were significantly greater in group 3 than in both group 1 (p=0.001 and p=0.002, respectively) and group 2 (p=0.002 and p=0.016, respectively). On finite element analysis, group 3 showed the highest peak von-Mises stress value (13.3 MPa) and compressive strain (3.2%) just above the tip of the blade within the femoral head. Meanwhile, groups 1 and 2 showed similar results on two values.

**Conclusions:**

Screw-blade hybrid type and blade type would be more effective in minimizing rotation instability of the proximal fragment in unstable basicervical IT fractures. However, varus collapse of the proximal fragment and cranial and axial migration within the femoral head were greater with blade type than screw-blade hybrid type.

## 1. Introduction

Basicervical intertrochanteric (IT) fractures are a specific type of trochanteric fracture that has rarely been reported as a separate entity [1–4]. In comparison with typical trochanteric fractures reported in earlier series, the fracture line crosses close to the base of the femoral neck and its junction with the IT region on radiographs [2, 5–7]. These fractures have not been well characterized in existing classification systems and are relatively rare. However, these are biomechanically extracapsular fractures and are usually treated with closed reduction and internal fixation [2, 4].

Because cephalomedullary nailing (CMN) has shown a clear advantage over the compression hip screw, the indications for CMN have been broadened greatly [8–10]. These expanded indications have led to increased CMN use for almost all peritrochanteric fractures, including basicervical fracture patterns [11–13]. Basicervical IT fractures may have inherent instability that makes treatment more difficult due to a relatively narrow cortical base of the proximal fragment and subsequent narrow contact area at the main fracture site along with insufficient cancellous interdigitation [14, 15]. Su et al. [7] suggested that basicervical fracture patterns may have greater biomechanical instability and noted that they collapsed more than typical IT fractures. More recently, Bojan et al. [16] reported that a basicervical fracture pattern is 1 of 3 variables associated with a high risk of screw cut-out. However, very little has been reported regarding biomechanical analysis or surgical outcomes focusing on basicervical IT fractures as a separate entity, especially when fixed with CMN.

To increase holding power for the proximal fragment and decrease rotational instability leading to fixation failure such as cut-out in unstable IT fractures, the fixation type of the femoral head has evolved and various types such as hip screw and helical blade have been used for CMN.

This study compared fixation stability of the proximal fragments and their mechanical characteristics in proximal femur models of unstable basicervical IT fractures fixed by CMN using 3 different types of the femoral head fixation. Biomechanical and finite element analyses were used to determine which type of the femoral head fixation is most effective and safe in this type of fracture.

## 2. Materials and Methods

### 2.1. Specimen Preparation

A total of 36 composite femurs with a customized density (Synbone LD 2386, Synbone AG, Switzerland) were used for this biomechanical study. All synthetic femurs were coated with a thin cortical layer and filled with low-density polyurethane foam corresponding to osteoporotic human bone. The models were 450 mm in length, with 122° neck shaft angle, 15° anteversion, 48 mm head diameter, and 12 mm canal diameter. Thirty-six femur models were divided into 3 groups to receive 3 different kinds of intramedullary (IM) nails: 12 Gamma 3 nails (Hip screw type; Stryker, Mahwah, NJ, USA) (Group 1), 12 Gamma 3 U-blade nails (Hip screw-blade type; Stryker) (Group 2), and 12 Proximal Femoral Nail Antirotation-II nails (PFNA-II, Helical blade type; DePuy Synthes, West Chester, PA, USA) (Group 3).

**Figure 1:**
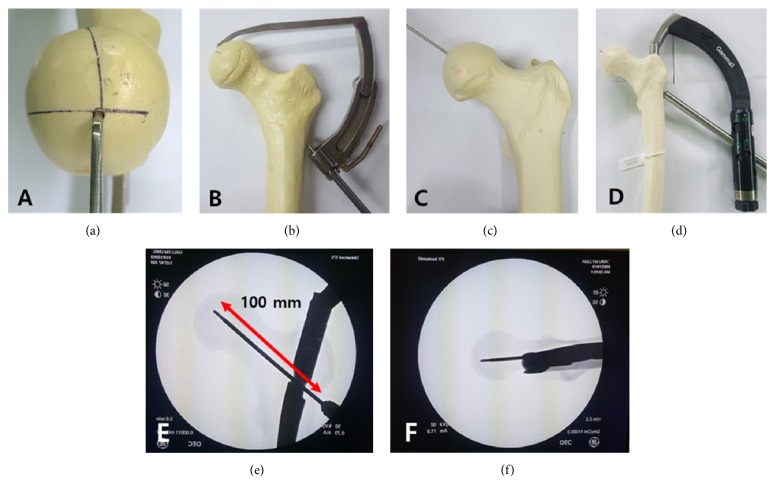
Illustration of procedure steps of inserting a hip screw in center-to-center within the femoral head using a posterior cruciate ligament tibial guide and targeting device of the nail after marking the center of the femoral head (a-d). Anteroposterior (e) and axial (f) views after placing the guide pin in center-to-center within the femoral head on fluoroscopy.

All implants were inserted by the same surgeon (JHY) with fluoroscopic guidance using a standard technique. First, we performed preliminary test using 2 specimens to exactly and uniformly place hip screw and blade in center-to-center within the femoral head. Because we planned to use CMNs with the centrum-collum-diaphyseal (CCD) angle of 125°, we could exactly insert a guide pin in center-to-center position under the confirmation of fluoroscopy using a posterior cruciate ligament (PCL) tibial guide set at 55° after marking the center of the femoral head in each specimen. Then, we pulled out part of the pin in the opposition direction from the femoral head to make CMN inserted and placed the tip of screw and blade about 10 mm beneath the apex of the femoral head at anteroposterior view using this guide pin and targeting device of nail after inserting CMNs into each specimen (Figure 1). At that time, we selected 100 mm as appropriate length of hip screw and blade. All specimens were fixed using nails with the same CCD angle (125°), length (170 mm), and diameter (12 mm), and hip screw or blade (100 mm) and distal locking screw (40 mm) of the same length were used in all specimens. The entry point of the nail was consistently placed at the tip of the greater trochanter (GT) in all cases. Hip screws or blades were consistently inserted in the center-to-center within the femoral head with the almost same tip-apex distance (TAD) in all specimens by using a PCL tibial guide and 100-mm screw or blade under fluoroscopy. A TAD less than 25 mm was confirmed at anteroposterior and axial radiographs in all specimens. Finally, TAD in all specimens ranged from 18 to 22 mm and TAD in each group was 19.9±0.6 mm in group 1, 19.8±0.7 mm in group 2, and 20.2±0.6 mm in group 3, respectively.

**Figure 2:**
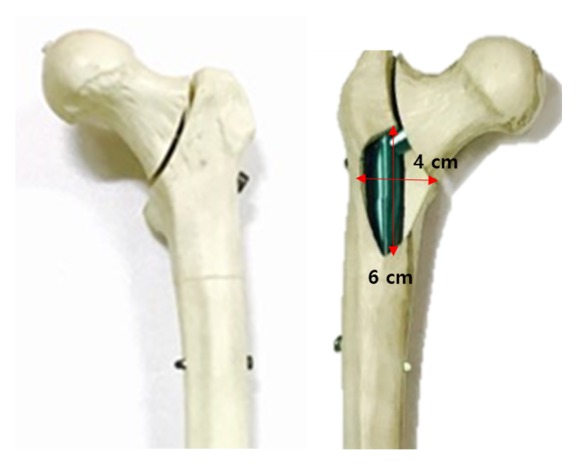
Unstable basicervical intertrochanteric fracture model fixed with a CMN. The main fracture line is located at the base of femoral neck and a uniformly-sized posteromedial fragment including the lesser trochanter is removed by an engraving machine based on a designed drawing on each specimen.

Unstable basicervical IT fractures corresponding to AO/OTA type 31-A2.2 were uniformly reproduced in all specimens fixed with the 3 kinds of nails, using an engraving machine (Shin-il Inc., Busan, South Korea) based on a designed drawing, and the fracture gap was set at 2 mm. The main fracture line was made at the base of the femoral neck and a uniformly-sized posteromedial fragment (6×4 cm) including the lesser trochanter (LT) was removed in all specimens (Figure 2).

The distal portion of all femoral bone models was cut at 30 cm distal from the GT tip using a customized 3-dimensional (3D) printed cutting frame, because an intact femur model might be broken at the femoral shaft during the loading test, and mounted on a steel square holder using resin [17]. Biomechanical testing of each specimen was performed in a vise at 25° adduction in the coronal plane and neutral in the sagittal plane to simulate one-legged stance [18–20].

**Figure 3:**
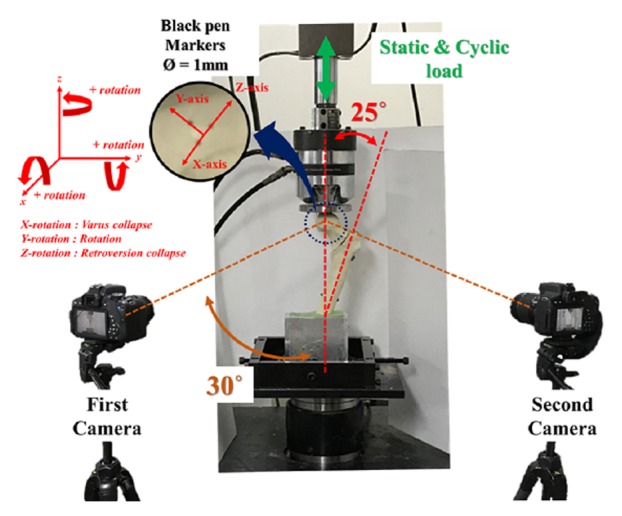
Illustration showing the test setup and 3D motion tracking system (stereophotogrammetry) for the proximal fragment including attachment of black pen markers. Two cameras were placed at 30 degrees to the center of the specimen to establish the experimental environment for stereophotogrammetry.

*Biomechanical Testing*. An MTS 858 Material Testing Machine (MTS Mini Bionix® Material Testing Systems, MTS Systems Corp., Eden Prairie, MN, USA) was used for loading, with a polished flat applicator that permitted free movement of the femoral head when loaded [21]. Three black markers 1 mm in diameter, which were not located collinearly, were placed at the front of the femoral head. Each specimen was positioned so that it could move within the 3D space defined by the calibration frame [22]. Two cameras were placed at 30° to the center of the specimen to measure the migration of the proximal fragment according to each axis (x-, y-, and z-axes) (Figure 3). The 3D linear transformation and angles before and after the experiment could be measured by applying direct linear transformation (DLT) for 3D coordinates using two cameras [23]. To confirm the accuracy of the 3D coordinates derived from this method, the linear transformation and rotation values were measured using physical objective system and the difference between each actual values and DLT values was calculated. The root-mean square (RMS) values of the difference were used to verify the accuracy of errors and the minimum measurement range in the system [24]. RMS errors for x-, y-, and z-axes were 0.13°, 0.25°, and 0.21°, respectively. Values measured lower than the RMS errors at each axis were excluded in the results.

Three pilot tests were performed to determine the optimal testing load in cyclic vertical loading on the synthetic bone models without implantation. This test was carried out by applying a load of 1400 N [25]. However, the final load value was set to 750 N because of the occurrence of femoral shaft fracture at 1100 to 1200 N [22]. The loading protocols were as follows. Initially, a preload of 100 N at a rate of 20 N/min was loaded to each specimen to ensure complete contact between the femoral head and the test equipment [26]. Next, the specimen was cyclically loaded, with vertical loads from 75 to 750 N at a rate of 2 Hz for 10,000 cycles [27, 28]. This cyclic loading was considered to simulate walking when fracture consolidation is assumed about 6 weeks after surgery [25]. After 5 minutes of relaxation, 2-dimensional photographs were taken for analysis of 3D migration of the proximal fragment. Then, x-rays were taken to measure migration of the screw or blade tip within the femoral head. Finally, the vertical load was continued at a speed of 10 mm/min until the construct failed, while recording load-displacement curves [29]. Failure was defined as fracture of the femoral neck and/or cut-out/cut-through, and/or implant failure, and/or displacement of the fragments in excess of 15 mm, and/or sudden drop of the load resistance observed at the load-displacement curve [17].

**Figure 4:**
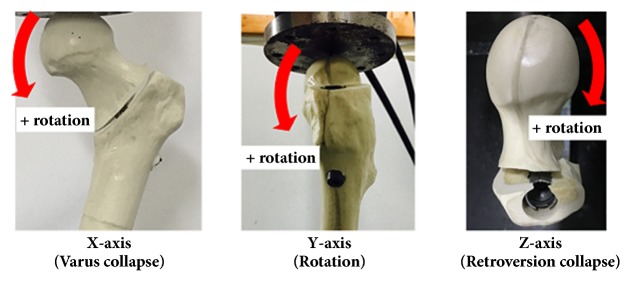
Definition of the migration direction for the assessment of 3-dimensional migration of the proximal fragment.

Migration of screw or blade tip within the femoral head in axial and cranial direction was measured in the frontal plane with a computer-aided design program (Rhinoceros 3, Robert McNeel & Associates, Seattle, WA, USA), using a radiograph in which the same ratio was adjusted by a plastic bar (155 mm length) before and after the experiment [17]. The migration of the proximal fragment was evaluated with an optical 3D motion tracking system (stereophotogrammetry) and Bryant angles [30, 31]. Calibration was performed to establish the laboratory coordinate system and to set up the calibration volume. Based on 3 markers on the femoral head, the extent of rotation, varus collapse, and retroversion collapse of the proximal fragment were measured along the three axes, before and after the experiment [24] (Figure 4).

### 2.2. Finite Element Analysis Study

We used a 3D femoral finite element model that was verified in previous studies [32]. This finite element model was reconstructed by geometry extraction and volume meshing (isotropic tetrahedral element) using Mimics Innovation Suite software (ver. 14.1, Materialise, Belgium) through two dimension slice images obtained from 1 mm-width cuts of computed tomography of a normal Korean adult femur [32]. The mechanical properties of cortical and cancellous bone, callus, and implants were applied, based on previous literature (Table 1) [33–35]. An unstable basicervical IT fracture corresponding to AO/OTA 31-A2.2 was reproduced with a fracture line with a radius of 110 mm at a distance of 76 mm from the tip of the femoral head on the coronal plane, and the thickness of the callus was set at 1 mm. The GT and LT were consistently removed. Three kinds of IM nails in this fracture model were positioned in the range of 20 to 24 mm TAD [36], and each postoperative finite element model was divided into group 1 (Gamma 3 nail, TAD: 22.9 mm), group 2 (Gamma 3 U-blade nail, TAD: 22.9 mm), and group 3 (PFNA-II, TAD: 22.5 mm).

**Figure 5:**
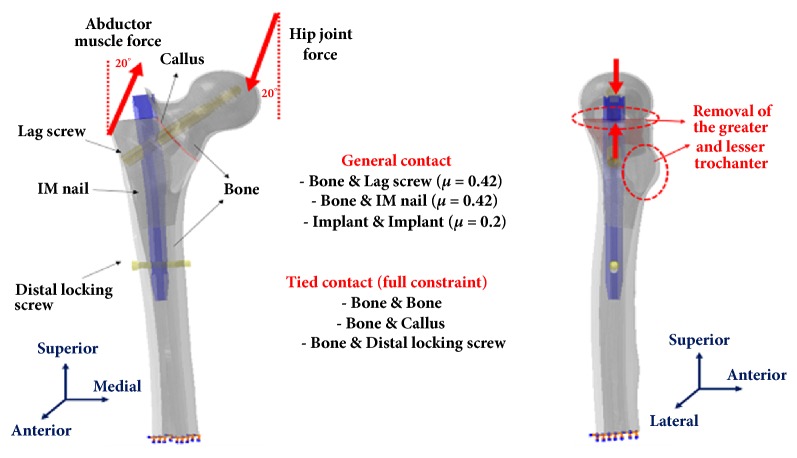
Illustration showing the finite element model reproducing an unstable basicervical intertrochanteric fracture and the loading and boundary conditions.

A finite element program (ABAQUS, Dassault, France) was used to analyze the biomechanical effect of 3 different designs of the femoral head fixation in an unstable basicervical IT fracture model. A hip joint force (2013.9 N) of 300% body weight (BW, 68.5 kg) was loaded on the femoral head and an abductor muscle force (671.3 N) of 100% of BW was loaded on the lateral surface of the GT [37]. Both force directions were set at 20° on the vertical axis in the frontal plane [37]. For the boundary conditions, the distal portion of the femur was fixed in all directions and the frictional contacts were defined by bone-implant interaction and implant-implant interaction. The friction coefficient was 0.42 for bone-implant interaction and 0.20 for implant-implant interaction [38]. Distal locking screw-bone interaction and bone-bone interaction (cortical to cancellous bone, bone to callus) were assumed to be fully constrained (Figure 5).

Biomechanical assessment of postoperative models fixed with IM nails with 3 different designs of the femoral head fixation was performed for the risk of cut-out or cut-through. To evaluate the risk of cut-out or cut-through within the femoral head, peak von-Mises stress (PVMS, MPa) and compressive strain (%) were measured for cancellous bone of the femoral head [38].

### 2.3. Statistical Analysis

After testing for normality (Kolmogorov-Smirnov test), one-way analysis of variance and a Tukey-Kramer post-hoc test (stiffness, failure load, cranial migration, and varus collapse) or Kruskal-Wallis test and a Mann-Whitney post-hoc test (axial migration, rotation, and retroversion collapse) were chosen to assess differences among the groups concerning the investigated variables. All statistical evaluation was completed using SPSS, V17 (SPSS Inc., Chicago, IL, USA) software. Values were reported as mean and standard deviation. Two-tailed p values less than 0.05 were considered statistically significant. However, a level of statistical significance after Mann-Whitney post hoc test was determined for p<0.017 (0.05/3) using Bonferroni's method. A priori power analysis was performed, based on studies in the literature as well as studies from our own laboratory, for which a sample size of 8 was sufficient to achieve 80% power at a significance level of 0.05. This sample size is also representative of similar studies in the literature.

## 3. Results

### 3.1. Stiffness and Failure Load

The mean stiffness at the load step of 900 N, which corresponds to the linear section within 5 mm deformation, did not reveal any significant differences among the 3 groups. However, group 3 showed the highest failure load (p<0.001) and there was no significant difference between groups 1 and 2 (p=0.488) (Table 2). Concerning the mode of failure, excessive displacement of more than 15 mm of the proximal fragments in the synthetic bone-implant constructs was observed in all but 2 of the 36 specimens. In the other 2 specimens, fracture of the proximal fragment occurred.

### 3.2. Migration of Screw or Blade within the Femoral Head

Group 3 showed 200% and 155% greater cranial migration of screw or blade within the femoral head compared to that in groups 1 and 2, respectively (p<0.001). Group 3 showed 450% and 175% greater axial migration compared to that in groups 1 and 2, respectively (p=0.003). Cranial and axial migration in group 3 was significantly greater than in both group 1 (p=0.001 and p=0.002, respectively) and group 2 (p=0.002 and p=0.016, respectively). There were no significant differences between groups 1 and 2 (Table 2).

### 3.3. Migration of the Proximal Fragment

The migration of the proximal fragment was measured according to 3 axes. The rotational change of the proximal fragment according to the axis of screw or blade (y-axis) was much greater in group 1 than in groups 2 and 3 (p=0.016 and p=0.007, respectively). There was no significance difference between groups 2 and 3 (p=0.865).

Varus collapse according to the x-axis revealed significant difference among the 3 groups (p=0.047). The extent was greater in group 3 than in group 2 (p=0.045). However, there were no differences between groups 1 and 2 and groups 1 and 3 (p=0.312 and p=0.631, respectively). There was no significant difference in retroversion collapse according to z-axis of the proximal fragment among the 3 groups (p=0.640) (Table 2).

### 3.4. Finite Element Prediction of Postoperative Models

**Figure 6:**
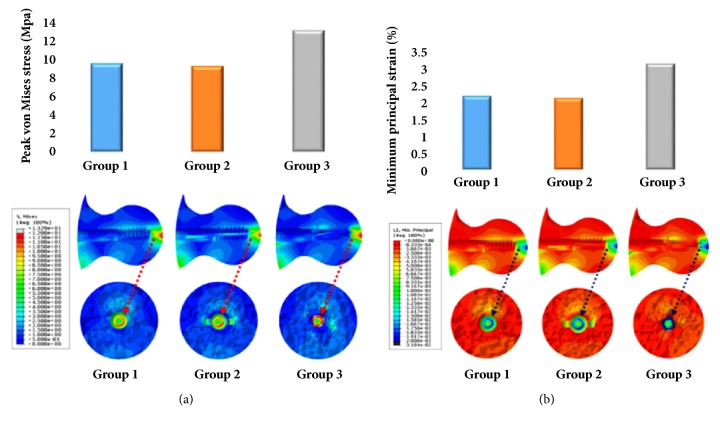
Stress distribution and compressive strain just above to the tip of lag screw within the femoral head among the 3 groups; (a) peak von Mises stress and (b) minimum principal strain.

Finite element models reproducing an unstable basicervical IT fracture fixed using 3 different IM nails were analyzed in terms of stress and compressive strain within the femoral head just above the tip of the screw or blade. While group 3 showed the highest PVMS value (13.3 MPa), group 1 and group 2 showed similar values (9.7 MPa vs 9.4 MPa) (Figure 6(a)). In addition, group 3 showed the highest compressive strain (3.2%) and groups 1 and 2 showed similar values (2.2% vs 2.1%) (Figure 6(b)). The highest risk of cut-out or cut-through was predicted for group 3.

## 4. Discussion

Basicervical proximal femoral fractures have rarely been reported as a separate entity [1–4]. These fractures may have inherent instability of the proximal fragment due to a narrow cortical base of the proximal fragment and subsequent narrow contact area at the main fracture site along with insufficient cancellous interdigitation compared to conventional IT fracture [14, 15]. Moreover, in unstable fracture types accompanied by trochanteric comminution and/or posteromedial fragments, the instability of the proximal fragment after CMN may increase even more.

Watson et al. [14] reported that all fixation failure after CMN for basicervical proximal femoral fractures developed in patients who had appropriate TAD <25 mm and anatomic or nearly anatomic reduction and suggested that factors other than surgical technique were probably responsible for the failures. More recently, Bojan et al. [16] reported that a basicervical fracture pattern is 1 of 3 variables associated with a high risk of screw cut-out. Therefore, the ideal implant for fixation of a basicervical IT fracture should withstand weight-bearing forces and maintain rotational stability of the short proximal fragment during bone healing. Firm fixation for the short proximal fragment will subsequently reduce the risk of fixation failure. Accordingly, the type of the femoral head fixation has evolved to increase the rotational stability and cut-out resistance within the femoral head.

However, to our knowledge, no biomechanical studies have compared IM nails with different types of the femoral head fixation (screw type, blade type, and hybrid type) in the treatment of unstable basicervical IT fractures. Therefore, we performed this biomechanical study to compare migration of the proximal fragment such as varus and retroversion collapse and rotation and the migration of screw or blade within the femoral head in unstable basicervical IT fracture models that were fixed using 3 IM nails with different types of the head fixation.

In our study, a direct linear transformation method (stereophotogrammetry) capable of perceiving specific coordinates in 3D space was used to measure 3D motion of the proximal fragment according to 3 axes. The extent of the migration of the proximal fragment could be measured with accuracy of 0.2°, and the minimum measured values for each axis were 0.2°, 0.4°, and 0.3°, respectively, which were greater than the accuracy of each axis; therefore, we believe that the extent of migration of the proximal fragment along each axis was relatively precisely measured using this method. During the preparation of each specimen, all hip screws or helical blades were inserted at the center-center position under the guidance of fluoroscopy in the head of all femur models to minimize rotation of the proximal fragment [39], and the fracture was created after inserting IM nails to reproduce anatomical reduction in all specimens. These procedures were performed to remove bias that was likely to affect our results.

In the current study, failure load was the greatest for PFNA-II, although the mean stiffness did not reveal any differences among the 3 groups. The structural stability of this nail construct was greatest, but we believe that this was only because of the different ingredients of the alloy material and the nail design, rather than directly related to fixation failure, especially in unstable basicervical IT fractures, considering our clinical data in which most fixation failures were caused by inherent instability of the proximal fragment. Meanwhile, rotation of the proximal fragment was greater with hip screw type (Gamma 3 nail) than blade type (PFNA-II) and hybrid type (Gamma 3 U-blade nail), and varus collapse of the proximal fragment was greater with PFNA-II than Gamma 3 U-blade nail although there was no significant difference in retroversion collapse of the proximal fragment among the 3 groups. Strauss et al. [27] reported that the helical blade of the trochanteric fixation nail was a biomechanically superior implant design compared to the standard sliding hip screw for fracture fixation in an unstable IT hip fracture. Knobe et al. [40] reported that there was no significant difference in biomechanical properties between the rotationally stable screw-anchor plate system (RoSA) with a novel screw-blade combination (hybrid type) and the PFNA (blade type) in an unstable IT hip fracture. Our results were similar with regard to rotational stability. Knobe et al. [40] also reported that migration of the implant tip with respect to the femoral head in cranial and axial direction showed no differences between the RoSA and PFNA. However, based on our results, cranial and axial migration of the helical blade within the femoral head were greater with PFNA-II compared to Gamma 3 and Gamma 3 U-blade nails, which was supported by finite element analysis. We believe that unstable basicervical IT fracture types with smaller and more unstable proximal fragments in our study accounted for these differences. Accordingly, our findings suggest that the hybrid screw-blade (Gamma 3 U-blade) is the most effective type in unstable basicervical IT fractures although large-cohort comparative clinical studies are needed.

The strengths of our study include the large number of specimens tested in 3 different constructs using different types of IM nails, the use of consistent synthetic femur models corresponding to osteoporotic bone in elderly patients, the creation of a consistent fracture pattern using an engraving machine, and the utilization of an optical 3D motion tracking system for measurement of the migration of the proximal fragment such as rotation and varus collapse with accuracy of 0.2°, and testing in static and cyclic loading phases. In addition, this study is the first to perform a biomechanical comparison of 3 IM nails with different types of the femoral head fixation in an unstable basicervical IT fracture, which is rare, but is prone to fixation failure.

There are also several limitations of the current study. One is the creation of an artificial fracture to simulate an unstable basicervical IT fracture. This artificial fracture does not truly reproduce the manner in which this fracture develops. Another limitation is that we could not accurately simulate all of the physiologic force components in the hip that are encountered during ambulation or normal activity. The biomechanical comparison performed in this study simply used axial loading to simulate the forces of a one-legged stance. Meanwhile, physiologic loading during activity is more complex and greater loads can occur in real situations.

## 5. Conclusions

On the basis of our results, screw-blade hybrid type and blade type would be more effective in minimizing rotation instability of the proximal fragment in unstable basicervical IT fractures. However, varus collapse of the proximal fragment and cranial and axial migration within the femoral head were greater with blade type than screw-blade hybrid type. Accordingly, considering these biomechanical comparative findings, screw-blade combination type may achieve better outcomes in fixation of unstable basicervical IT fractures, especially in osteoporotic elderly patients. However, our results will have to be substantiated by further biomechanical and clinical trials.

## Figures and Tables

**Table 1:** Mechanical properties of the cortical, cancellous and callus bones, and implants for the current finite element model.

Part	Component	Young's modulus (MPa)	Poisson's ratio
Bone	Cortical bone	17,000	0.3
	Cancellous bone	445	0.2
	Callus	20	0.3
Implant	Gamma 3 (TI6Al4V)	113,800	0.342
	Gamma 3 U-blade (TI6Al4V)		
	PFNA-II (TI6Al7Nb)	110,000	0.35

**Table 2:** Results of the biomechanical test series for three different IM nails (Mean ± SD).

Variables	Group 1	Group 2	Group 3	P-value
	(n = 12)	(n = 12)	(n = 12)	
Stiffness (N/mm)	241 ± 53	251 ± 31	238 ± 43	0.260^*∗*^
Failure load (N)	1720 ± 354	1892 ± 299	2332 ± 363	**<0.00**1^**∗**^
Cranial migration (mm)	0.5 ± 0.3	0.6 ± 0.5	1.5 ± 0.8	**<0.00**1^**∗**^
Axial migration (mm)	0.2 ± 0.1	0.4 ± 0.4	1.1 ± 0.8	**0.00**3^†^
Rotation (degrees)	4.6 ± 3.0	2.3 ± 1.6	2.2 ± 1.1	**0.01**3^†^
Varus collapse (degrees)	1.3 ± 0.9	0.7 ± 0.5	1.7 ± 0.8	**0.04**7^**∗**^
Retroversion collapse (degrees)	2.1 ± 1.6	1.4 ± 1.0	1.9 ± 1.2	0.640^†^

^*∗*^One-way analysis of variance.

^†^Kruskal-Wallis test.

## Data Availability

The data used to support the findings of this study are included within the article.
